# Polymer Nanoparticles as Functional Fillers for Composite Electrolytes for Batteries

**DOI:** 10.1021/acs.iecr.6c00977

**Published:** 2026-06-19

**Authors:** Alejandro Herranz Berzosa, Gabriele Lingua, Leire Unanue, Alejandro J. Müller, Maria Forsyth, Jose Ramon Leiza, David Mecerreyes

**Affiliations:** † POLYMAT, 160665 University of the Basque Country UPV/EHU, Avenida Tolosa 72, 20018 Donostia-San Sebastian, Spain; ‡ IKERBASQUE, Basque Foundation for Science, Plaza Euskadi 5, 48009 Bilbao, Spain

## Abstract

Different nanofillers, such as inorganic nanoparticles or nanofibers, are commonly used to design composite electrolytes with superior mechanical and electrochemical properties for batteries. As an alternative, we review here the recent use of polymeric nanoparticles obtained by drying nanolatexes synthesized by emulsion polymerization. These polymeric nanoparticles offer key advantages vs inorganic nanoparticles, such as scalable synthesis, controllable chemical nature, size control, easy surface functionalization, low density, and excellent dispersion within polymeric matrices. First, we will discuss the synthetic method of sulfonamide-functional poly­(methyl methacrylate) nanoparticles (LiNPs) in order to control the particle chemical nature, surface functionality, and size between 20 and 100 nm. Next, we will show how these polymeric nanoparticles can be used to prepare composite electrolytes with improved mechanical properties, high ionic conductivity, and/or lithium single-ion conduction. Finally, the excellent performance of the different composite electrolytes in both lithium- and sodium-metal batteries will be shown.

## Graphical abstract

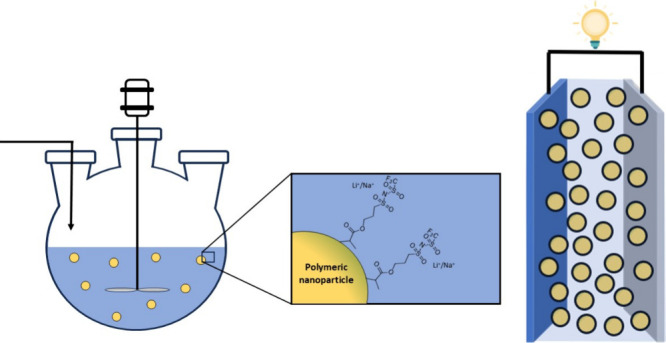

## 1 Introduction

Solid electrolytes that show fast ion conduction, while avoiding the common drawbacks of liquid electrolytes such as flammability and leakage, are actively being sought to improve the performance, conformability, and safety of batteries.^1^ Among the different types of solid electrolytes, composite electrolytes based on a combination of polymers, inorganic nanoparticles or nanofibers, organic solvents, ionic liquids, and/or salts are the current choice for state-of-the-art solid-state batteries.^2^ Composite electrolytes overcome some of the limitations shown by conventional solid polymer electrolytes such as poor mechanical properties, low ionic conductivity, and low lithium transference number or interfacial stability.

Conventional composite electrolytes are prepared by mixing a polymer electrolyte matrix such as poly­(ethylene oxide) with a lithium salt and nanosized fillers such as inorganic nanoparticles (e.g., SiO_2_, TiO_2_) or other nanofibers.^3^ Although inorganic oxide nanoparticles are the most common fillers, in the past few years, there has been high interest in functional porous nanomaterials such as MOFs, zeolites, or silica mesoballs.

Here, the inorganic nanoparticles help to improve the mechanical properties of the polymer electrolyte and the ionic conductivity due to the nanostructuration effect and the fast conduction pathway generated at the particle–polymer interphase, which facilitates stable interfaces.^4^ A critical point in these systems is that the surface functionalization of the inorganic nanoparticle is key for improving its dispersibility while promoting the ion conduction.^5^ Interestingly, the organization of the inorganic nanoparticles within nanostructured polymer systems is key to tune their ionic conducting properties, as pioneered by Balsara in the case of block copolymers or Glynos and co-workers in miktoarm star architectures.^6,7^ On the other hand, composite electrolytes can also be fabricated by using inorganic nanoparticles to solidify or jellify liquid electrolytes, i.e., organic carbonates, glymes, or ionic liquids using inorganic and hairy nanoparticles.^8,9^

However, it is well-known that functionalization of inorganic nanoparticles or nanofibers is not an easy task. The synthesis of functionalized inorganic nanoparticles on a large scale presents several challenges, including scalability, specific surface chemistries for the grafting process, and tedious purification procedures.^10–12^ Surprisingly, much less attention has been paid to the use of polymeric nanoparticles as nanofillers in composite electrolytes. In principle, polymer nanoparticles offer several advantages over inorganic nanoparticles, such as a scalable synthesis, low density, good compatibility with other polymer matrices or organic electrolytes and easy surface functionalization. In this article, we showcase our recent efforts in the development of functional polymer nanoparticles as nanofillers in composite electrolytes for batteries. First, we will present a scalable synthetic method for functional polymeric nanoparticles via emulsion polymerization. Next, we will show how these polymeric nanoparticles can be used to prepare new types of all-polymer composite electrolytes and nanocomposite gel electrolytes with high ionic conductivity and lithium single-ion conduction. Finally, the potential for application of these nanocomposites in both lithium and sodium batteries will be shown.

## 2 Synthesis of Nanolatexes as a Route to Obtain Functional Polymeric Nanoparticles

Emulsion polymerization is a well-known industrial polymerization method for producing polymer latexes consisting of polymer particles stabilized in water by surfactant(s), with controllable and monodispersed sizes between 50 and 500 nm.^13^ Surface functionalization of the nanoparticles with ionic groups is carried out simply by including ionic monomers or polymerizable surfactants in the reaction formulation without the need for additional steps. Interestingly, Asua’s group demonstrated that the emulsion polymerization conditions could be pushed toward the synthesis of nanolatexes with particle sizes as small as 13 nm using relatively low surfactant concentration and high solids content.^14^ By using a theory- guided strategy for nanolatex synthesis, they optimized the required emulsion polymerization conditions such as surfactant concentration, solids content, monomer feeding rate, radical generation rate, polymer hydrophilicity, or temperature. As mentioned before, a second advantage of emulsion polymerization is that the chemical nature of the polymer particles and their functionality can be easily modified by the choice of the monomers. In most commercial latexes used in paints or adhesives, the functionalization of the polymer particles is typically carried out using acrylic acid in the latex formulations, which also enhances colloidal stability. As another possibility, Asua and Tomovska also showed that ionic monomers such as styrenesulfonate can be incorporated into polymer latexes in a surfactant-free process.^15^ The advantage of this process is that there is no need for additional cleaning steps to obtain the pure ionic functional particles without surfactant contamination.

Inspired by these fundamental works of Asua’s group, we designed a synthetic route toward polymeric nanoparticles to be used in composite electrolytes for batteries. In our work, we have chosen to surface functionalize the polymer nanoparticles with an anionic sulfonamide group by including in the latex formulation the comonomer lithium 1-(3-(methacryloyloxy)­propylsulfonyl)-1-(trifluoromethylsulfonyl)­imide (LiMTFSI) developed by Shaplov.^16^ Nowadays, this monomer is the most popular choice for obtaining state-of-the-art lithium single-ion conducting polymer electrolytes due to the high charge delocalization of the sulfonamide anion, which benefits cation mobility. In a pioneering work, Porcarelli developed the synthesis of sulfonamide functional cross-linked poly­(methyl methacrylate) polymer nanoparticles (LiNPs) by using a surfactant free semibatch emulsion polymerization process.^17^ It is worth noting that the polymer nanoparticles were cross-linked to ensure that their size is maintained after drying the latexes and that the polymer nanoparticles become insoluble. In this process, the ionic monomer LiMTFSI provided functionality on the surface of the polymer particles, stability to the latex and control of the polymer particle size. Thus, by adjusting the reaction conditions, spherical polymer nanoparticles with sizes between 95 and 200 nm were obtained with varying lithium sulfonamide groups in its surface. Although this process showed that we could synthesize the desired nanoparticles using emulsion polymerization, we wanted to go a step further and decrease their size.

For this reason, A. Gallastegui investigated the effect of adding a surfactant to the previous latex formulation.^18^ Thus, in order to synthesize very small NPs, we introduced 5 wt % of lithium dodecyl sulfate as a surfactant. In this case, after the emulsion polymerization process, transparent polymer latexes were obtained, indicating the production of a nanolatex with small-size polymer nanoparticles. One of the limitations of using surfactants is the need for a purification step by dialysis before drying the nanolatex to obtain clean, cross-linked nanoparticles. Interestingly, the polymer nanoparticles showed very small sizes between 20 and 25 nm. Furthermore, the LiMTFSI comonomer composition could be varied without a detrimental effect on the size, allowing the lithium sulfonamide content of the nanoparticles to be tuned. As shown by dynamic light scattering (DLS) measurements and TEM images, the particles exhibited a small spherical size and low dispersity.

This developed synthetic procedure shown in Figure 1 is quite versatile and allows modification of several aspects of the NPs, such as the chemical nature of the cation (Na vs Li) or the composition of the methacrylic polymer backbone. Thus, in a following work, Stilgliano showed that a similar procedure could be used to synthesize cross-linked poly­(methyl methacrylate) nanoparticles functionalized with a sodium sulfonamide group. In this case, the sodium version of the previous sulfonamide methacrylic monomer NaMTFSI was used as a comonomer and sodium dodecyl sulfate as the surfactant using a similar procedure.^19^ In the sodium case, as compared to the lithium one, slightly bigger polymer nanoparticles of 50 nm size were obtained as compared to the lithium ones under the same reaction conditions. Related to the chemical composition of the polymer particle, Herranz showed that the polymer backbone of the nanoparticle could be varied from poly­(methyl methacrylate) and include other (co)­monomers such as styrene or poly­(ethylene glycol) methacrylate while maintaining a small size around 20 nm.^20^

**Figure 1:**
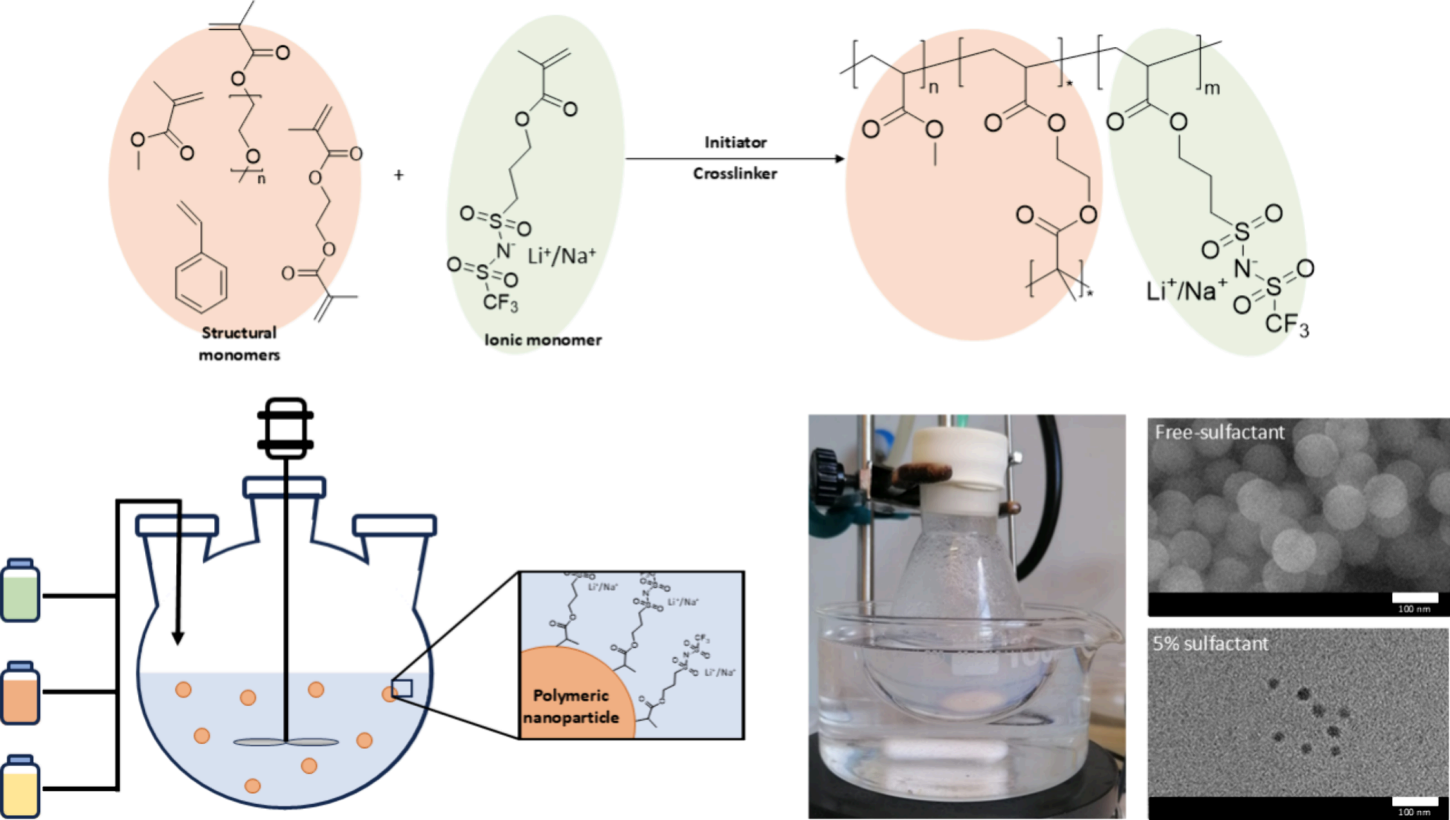
Synthetic route toward sulfonamide functional cross-linked poly­(methyl methacrylate) nanoparticles by semi-batch emulsion polymerization. Pictures of the obtained nanolatex and TEM pictures of nanoparticles obtained in a surfactant-free process and with 5 wt % of surfactant. *Reproduced from ref* ^20^. *Copyright © 2025 American Chemical Society. Reproduced from ref* ^18^. *Copyright © 2023 Gallastegui et al. Small Science published by Wiley-VCH GmbH. Reproduced from ref* ^17^. *Available under a CC-BY 4.0 license. Copyright © 2021 Porcarelli et al. Published by American Chemical Society.*

## 3 Polymeric Nanoparticles as Fillers in Composite Electrolytes for Batteries

In this section, the potential of these functional polymer nanoparticles in composite solid electrolytes for lithium batteries is discussed. Two types of composites were investigated. In the first case, the particles were mixed with a polymer electrolyte based on poly­(ethylene oxide), forming an all-polymer nanocomposite. In the second case, the nanoparticles were combined with a solvent or liquid electrolyte to prepare the nanocomposite gel electrolytes.

### 3.1 All-Polymer Nanocomposite Electrolytes

Although its ionic conducting properties were discovered more than 50 years ago, poly­(ethylene oxide) (PEO) polymer electrolytes are still considered the gold standard materials for lithium metal solid-state batteries.^1^ However, the ionic conductivity of PEO does not reach usable levels (>10^–4^ S cm^–1^) until melting of the polymer crystalline regions (*T*_m_ > 60 °C). At these high operating temperatures, PEO mechanical properties are lost, and to circumvent this issue, various strategies have been proposed, such as PEO cross-linking, blending with a rigid polymer, or the use of block copolymers. As a popular alternative, the mechanical properties of PEO polymer electrolytes can also be improved by preparing composites with nanofillers or nanosized inorganic materials (SiO_2_ or TiO_2_ nanoparticles or nanofibers). As mentioned in the introduction, these nanoparticles also show a beneficial effect on the ionic conductivity with the right surface functionalization and distribution within the PEO matrix.^21^

As an alternative to the inorganic nanoparticles, we proposed using of the LiNP as nanofillers in PEO polymer electrolytes. Two types of PEO/NP nanocomposites were investigated. The first one is the conventional nanocomposite composed of PEO, a lithium salt, and the nanoparticles. In our initial work, by L. Porcarelli, with the 100 nm nanoparticles, showed that when mixed with poly­(ethylene oxide) and lithium bis­(trifluoromethane)­sulfonimide (LiTFSI/PEO), the particles induce a significant stiffening effect (*E*′ > 10^6^ Pa vs 105 Pa at 80 °C), while the membranes retain high ionic conductivity (σ = 6.6 × 10^–4^ S cm^–1^ at 80 °C).^17^

The second type of nanocomposite polymer electrolyte is composed solely of PEO and LiNPs without additional lithium salt. In this case, the lithium conduction is only due to the lithium ions coming from the lithium sulfonamide functional groups of the nanoparticles. Since the sulfonamide is attached to the polymer backbone, the composite should show lithium single-ion conduction, as evidenced by high lithium transference numbers. It is worth noting that lithium single-ion conductors are actively investigated nowadays due to their ability to alleviate the lithium dendrite formation during battery cell operation.^22^

Thus, Olmedo-Martinez reported the first salt-free all-polymer nanocomposite solid electrolyte composed of lithium sulfonamide-functionalized poly­(methyl methacrylate) nanoparticles of very small size (20–30 nm) mixed with poly­(ethylene oxide) (PEO).^23^ The all-polymer nanocomposites showed a very good distribution of the polymer nanoparticles even at high contents (50 LiNP wt %) as observed by TEM. This is a very important fact, confirming the excellent dispersion of the polymer nanoparticles within a polymer matrix, which is not easy to achieve in the case of inorganic analogues. Indeed, Olmedo-Martinez investigated in detail how the crystallinity of PEO was affected by the presence of the LiNPs. Using dynamic mechanical thermal analysis (DMTA), it was shown that the LiNPs strengthen the PEO nanocomposite. The highest ionic conductivity value for the PEO 50 wt % LiNP nanocomposite at 80 °C was 1.1 × 10^–5^ S cm^–1^. This lower ionic conductivity compared to the salt containing nanocomposite was expected, but remarkably the salt-free nanocomposite showed lithium single-ion conducting behavior characterized by a lithium transference number of 0.68.

In a subsequent work, Jiang et al. and Olmedo went one step further and investigated how the LiNPs could be ordered within the interlamellar regions of semicrystalline poly­(ethylene oxide) (PEO).^24^ Specifically, lithium sulfonamide functional polymeric methacrylic nanoparticles (NPs) with particle sizes of 25 nm were aligned within a PEO matrix by controlling the crystallization rate of PEO. This ordering was observed by using transmission electron microscopy (TEM) and small-angle X-ray scattering (SAXS). Interestingly, the alignment of the NPs results in an eight-fold increase in the ionic conductivity of the nanocomposite polymer electrolyte at room temperature, maintaining lithium single-ion conducting behavior.

All in all, these results confirmed that polymeric nanoparticles are an alternative to inorganic ones in the development of PEO nanocomposite electrolytes, as shown in Figure 2. The polymeric nanoparticles exhibit excellent dispersibility within the polymer matrix, and their facile surface functionalization offers unique opportunities such as the formation of single-ion-conducting polymer electrolytes.

**Figure 2:**
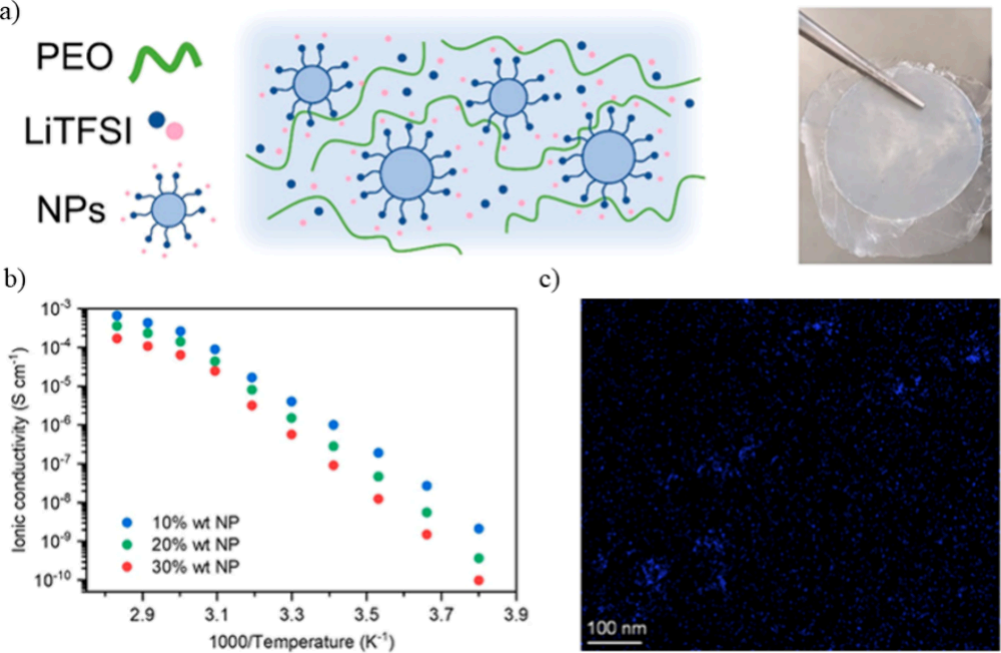
(a) Schematic representation of a PEO nanocomposite electrolyte containing polymer nanoparticles and a lithium salt, (b) ionic conductivity of a PEO/LiTFSI electrolyte containing 100 nm polymer nanoparticles, (c) TEM image of a PEO/polymer NP nanocomposite. *Reproduced from ref* ^17^. *Available under a CC-BY 4.0 license. Copyright © 2021 Porcarelli et al. Published by American Chemical Society. Reproduced from ref* ^23^. *Available under a CC-BY-NC-ND 4.0 license. Copyright © 2024 Olmedo-Martinez et al. Published by American Chemical Society.*

#### 3.1.1 Nanocomposite Gel Electrolytes

Nanocomposite gel electrolytes are semisolid electrolytes that combine the mechanical and structural properties of polymers with the high ionic conductivity of organic solvents or ionic liquid electrolytes. When gel electrolytes are combined with lithium single-ion conductors, a unique combination of high transference number and high ionic conductivity near room temperature can be achieved. For this reason, the lithium sulfonamide polymer nanoparticles are particularly interesting for designing nanocomposite electrolytes in combination with different organic solvents typically used in battery electrolytes without the need for additional lithium salts. This concept was first demonstrated with the lithium sulfonamide 100 nm nanoparticles mixed with propylene carbonate in different ratios up to a maximum of 60 wt % LiNP content (Figure 3a). The obtained gel electrolytes were mechanically robust and exhibited an Arrhenius-type conductivity with a lithium transference number close to unity. The electrolyte with the lowest polymeric nanoparticle loading, 20 wt %, showed the highest ionic conductivity, 2.8 × 10^–4^ S cm^–1^ at 85 °C, which is a very high value for a lithium-single ion conductor.^13^

**Figure 3:**
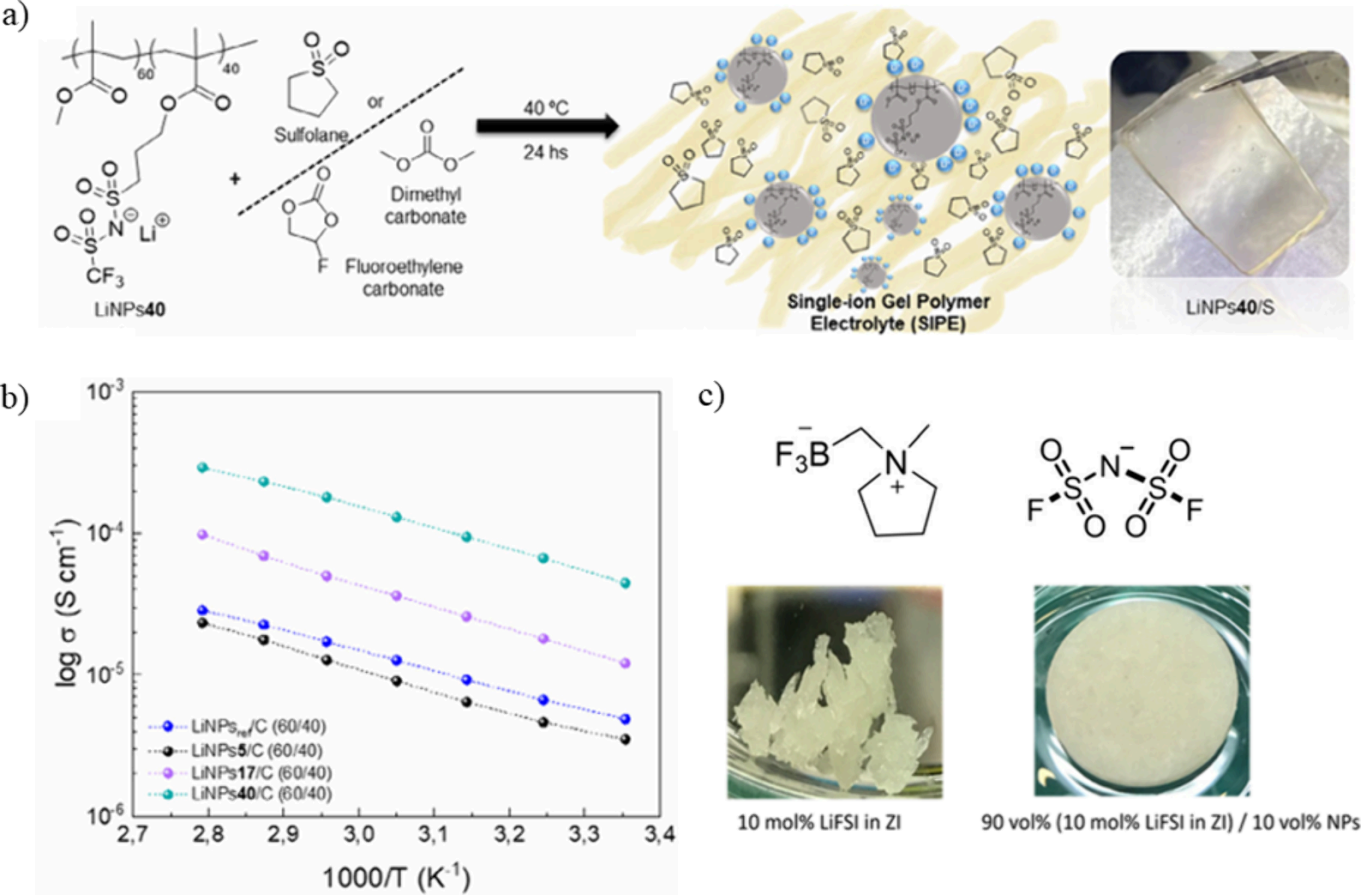
(a) Single-ion conducting lithium polymer electrolyte preparation employing LiNPs and sulfolane/carbonates plasticizers. (b) Ionic conductivity versus temperature of nanocomposite gel electrolytes with different plasticizers. (c) Picture of zwitterionic organic ionic plastic crystal electrolyte and the nanocomposite including 10 wt % polymer NP. *Reproduced from ref* ^18^. *Copyright © 2023 Gallastegui et al. Small Science published by Wiley-VCH GmbH. Reproduced from ref* ^27^ *with permission from the Royal Society of Chemistry.*

Next, we investigated the effects of the nanoparticle size and the LiMTFSI comonomer content on the ionic conductivity of the nanocomposite gel electrolytes. The polymer electrolytes prepared with the “small” nanoparticles (25 nm) present three times higher ionic conductivity than the same nanocomposite electrolyte prepared with the “big” (100 nm) polymer nanoparticles. This huge difference in ionic conductivity justifies our synthetic efforts toward small nanoparticles. We hypothesized that small nanoparticles had a large surface area and, therefore, a greater exposure of the sulfonamide lithium within the more mobile plasticized regions. On the other hand, the ionic conductivity of the nanocomposite gel electrolytes shows a higher value when the amount of lithium sulfonamide content increases, i.e. an order of magnitude increase in ionic conductivity when 40 mol % of the lithium monomer is present in the copolymer versus only 5 mol %. However, the increase in ionic conductivity cannot be merely due to an increase in the number of Li^+^ ions, as this is only a factor of 8, whereas the conductivity increase is more than 1 order of magnitude.

The chemical nature of the organic solvent or plasticizer used to prepare the gel nanocomposites has an important role in their properties. For instance, when sulfolane is used as a plasticizer instead of propylene carbonate, the membrane became more manageable and free-standing, while the ionic conductivity decreases as compared with the cyclic carbonate case, where the membranes are softer, albeit more conductive. We also extended this concept to the case of sodium sulfonamide functional nanoparticles. In this case, different solvents were investigated at 50 wt % to prepare nanocomposite gel electrolytes by mixing the NPs with diglyme, sulfolane, propylene carbonate, and different ionic liquids. The nature of the plasticizer deeply influenced the sodium coordination shell, impacting the properties of the membranes. The highest ionic conductivity was achieved using sulfolane, showing predominantly a monodentate type of sodium coordination.

Lastly, the effect of polymer nanoparticle’s chemical nature was also investigated by incorporating different comonomers such as styrene (rigid and apolar) and poly­(ethylene glycol)­methacrylate (soft and polar) into the 25 nm poly­(methyl methacrylate) nanoparticles. Nanocomposite gel electrolytes were investigated in combination with sulfolane. In both cases, the ionic conductivity value was lower than in the original poly­(methyl methacrylate) nanoparticles, however, the inclusion of styrene showed a beneficial effect on the mechanical modulus and on its performance as a solid electrolyte with lithium metal anodes.

Finally, it is also worth mentioning that the beneficial role given by the polymer nanoparticles was also used to design novel ionic nanocomposite gel electrolytes including organic ionic plastic crystals (OIPCs) such as zwitterionic plastic crystals (Figure 3b,c). OIPCs are promising materials for the development of solid-state electrolytes for next-generation energy storage devices with improved safety. Incorporating polymeric nanoparticles (NPs) into OIPC matrices can create solid-state electrolytes with increased mechanical stability and enhanced ionic conductivity.^25^ In this series of work, in collaboration with Pringle’s group, self-standing, conductive zwitterionic-based composite materials were developed by combining lithium functionalized polymer nanoparticles with different OIPCs such as *N*-methyl-N-ethylpyrrolidinium bis­(trifluoromethanesulfonyl)­amide as well as zwitterionic ones such as trifluoro­((1-methylpyrrolidin-1-ium-1-yl)­methyl)­borate. The further addition of either lithium salt or solvent was used to optimize the ionic conductivity and/or transference number of the nanocomposite electrolytes. The effect of the formation of interfaces and interfacial regions between the OIPC and the polymer nanoparticle on the thermal stability, ion transport, morphology, and ion dynamics were studied in detail using DSC, polarized optical microscopy, and solid state NMR.^26^ The disruption of the crystalline lattice of the OIPC after adding the polymer nanoparticles leads to the formation of disordered interfacial regions with increased ionic conductivity and ion dynamics of lithium. Additional lithium salt doping of the polymer-based composite further doped the OIPC and increased the ionic conductivity and lithium transport.^27^

## 4 The Use of Polymer Nanoparticles in Composite Electrolytes for Lithium and Sodium Batteries

Most of the nanocomposite electrolytes described in Section 3 show sufficient ion transport and mechanical properties to be further characterized in metal anode-based batteries. In most of the works, our composite electrolytes were cycled in lithium cells to evaluate the stability against the lithium metal electrode and the resistance to dendrite growth. Indeed, we first demonstrated that the stable cycling of lithium could be achieved with different gel nanocomposite electrolytes. As an illustrative example, the composite electrolyte formed by a zwitterionic OIPC and a LiFSI lithium salt showed very stable Li deposition and dissolution, with cycling longevity demonstrated for up to 500 h. This was attributed to the high ionic conductivity and improved lithium transference number given by the polymer NPs.

On the other hand, sodium metal anode cycling in a symmetric cell configuration was also shown using a nanocomposite gel electrolyte based on the sulfonamide sodium polymer nanoparticles with a hybrid electrolyte composed of an ionic liquid and sulfolane or propylene carbonate (Figure 4). Here, it was shown that the use of ILs as plasticizers proved to be more beneficial for SEI formation and its evolution during cell cycling. These preliminary results demonstrate the potential of using nanocomposite gel electrolytes as soft solid electrolytes for Na metal batteries.

**Figure 4:**
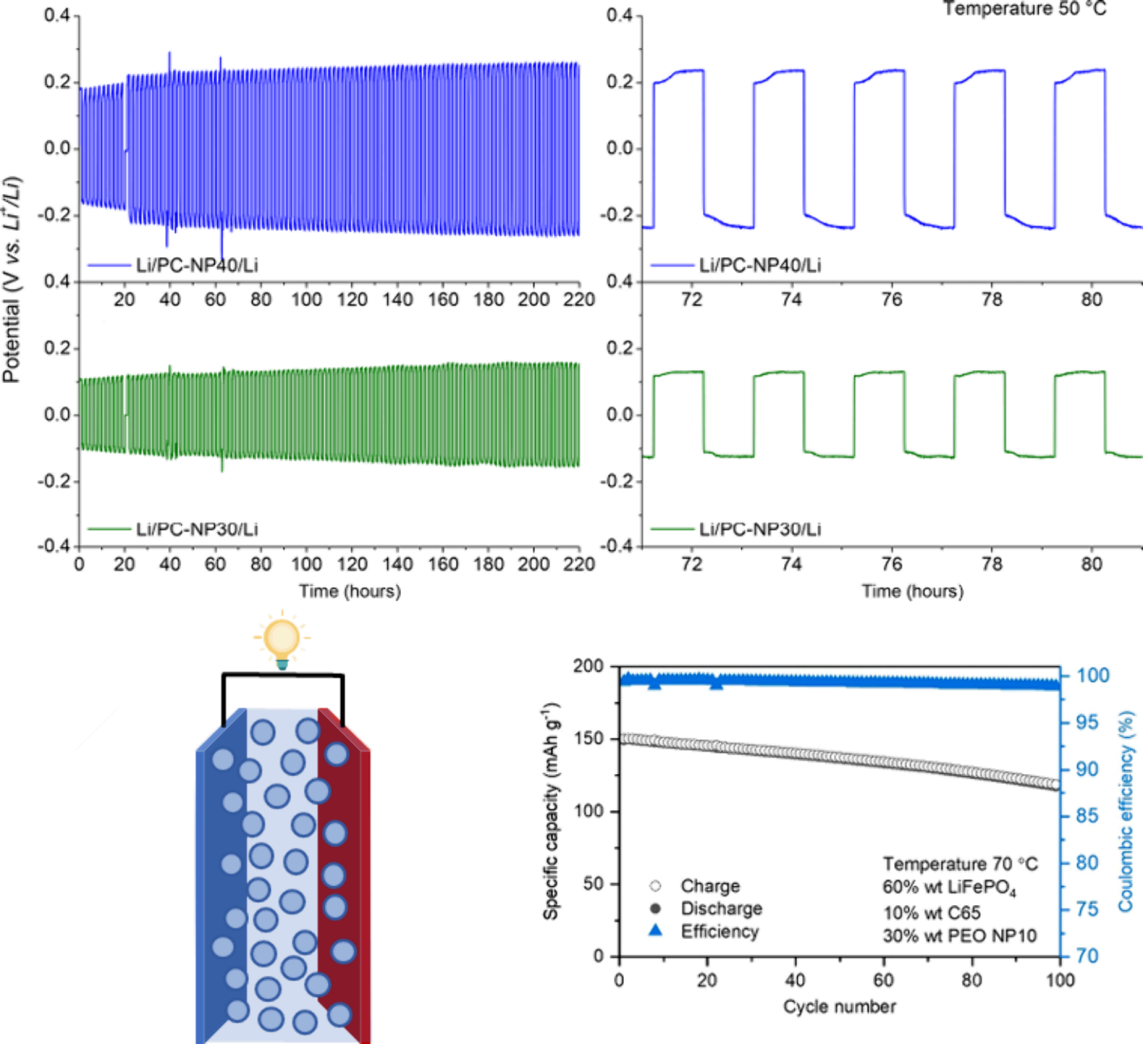
Upper graph, lithium symmetrical cell cycling of nanocomposite gel electrolytes based on propylene carbonate and polymer nanoparticles plot of the overpotential as a function of time cycled at 0.1 mA cm^–2^ at 50 °C. Lower graph, plot of specific capacity versus cycle number of an all polymer nanocomposite Li/PEO-NP10/LiFePO4 cell at 70 °C. *Reproduced from ref* ^17^. *Available under a CC-BY 4.0 license. Copyright © 2021 Porcarelli et al. Published by American Chemical Society.*

The nanocomposite gel electrolytes were also tested in full battery cells in the presence of different cathodes, showing promising performance. For instance, the all-polymer nanocomposites were tested using LiFePO_4_ as cathode materials. Figure 4 also shows the results obtained with a PEO nanocomposite electrolyte including LiTFSI salt and shows the long-term cycling performance at 70 °C at a constant current rate of C/10. After 100 cycles, the capacity retention was found to exceed 83% of the initial capacity, 150 mA h g^–1^, and the Coulombic efficiency approached 100%, demonstrating excellent cell performance of the composite electrolytes. Interestingly, similar results were also obtained with a salt-free PEO nanocomposite system, which shows lower ionic conductivity but higher lithium transport number.

Furthermore, the use of the nanocomposite gel electrolytes in full cells was also shown using NMC cathodes. NMC111 cathodes could be cycled with a sulfolane-based nanocomposite gel electrolyte at a current density of C/20, reaching a capacity of 121.6 mAh g^–1^ with a Coulombic efficiency close to 100% with good capacity retention in the first cycles. As the final example, the nanoparticles based on a styrene-methacrylate copolymer mixed with a sulfolane electrolyte allowed stable cycling in a lithium-metal cell in combination with NMC622 cathodes. The nanocomposite gel electrolyte demonstrated excellent C-rate performance along with high specific capacities of ∼160 mAh g^–1^ at C/20 with a minimal capacity fading over extended cycling and Coulombic efficiency of up to 99.73%.

## 5 Summary and Future Challenges

In this Mini-Review, we showcase our recent work on the use of polymeric nanoparticles as high-performance nanofillers for composite electrolytes for batteries. The developed synthetic method provides functional poly­(methyl methacrylate) polymer nanoparticles by drying nanolatexes obtained by an optimized emulsion polymerization process. The polymer nanoparticles offer some advantages over inorganic ones such as their scalable synthesis, controllable chemical nature (methyl methacrylate, styrene, ethylene glycol methacrylate, etc.), size control between 20 and 200 nm, easy surface functionalization with lithium or sodium sulfonamide groups, and excellent dispersion within polymeric matrices such as PEO.

These polymeric nanoparticles can be used to prepare composite electrolytes with improved mechanical properties, high ionic conductivity, and/or lithium single-ion conduction. Two types of composites can be distinguished. In one case, the so-called all-polymer nanocomposite electrolyte was used, where the nanoparticles were mixed with a poly­(ethylene oxide). We showed that both high-performance PEO nanocomposites with improved mechanical properties and ionic conductivity could be prepared, as well as original single-ion-conducting polymer electrolytes without the need for additional lithium salt.

Furthermore, the nanoparticles could be combined with solvents such as propylene carbonate or sulfolane or with an ionic liquid or organic ionic plastic crystal to prepare nanocomposite gel electrolytes. This type of gel electrolyte showed great performance when a zwitterionic-based electrolyte was used in combination with the polymer nanoparticles. Furthermore, new possibilities such as 3D printing of the composites using direct ink writing methods were shown, which can be used in the future as a new method to fabricate batteries.

Finally, our exploratory works have shown the potential use of the different composite electrolytes in different high-energy battery configurations, including different cathodes such as LiFePO_4_ or NMC and anodes such as lithium or sodium metal. Our current efforts are devoted to scaling up the synthetic process of the LiNPs and to testing the performance of the composite electrolytes in battery prototypes. To conclude, the future challenge will be to demonstrate the potential economic and environmental benefits of the polymer nanoparticles shown here in the battery industry.

## Notes

This article is dedicated to Prof. J.M Asua. His pioneering works in the area of polymer nanolatexes and particle surface functionalization by emulsion polymerization process enabled to prepare the polymer nanoparticles used in our works.

## References

[1] Song Z., Chen F., Martinez-Ibañez M., Feng W., Forsyth M., Zhou Z., Armand M., Zhang Z. (2023). A Reflection
on Polymer Electrolytes for Solid-State Lithium Metal Batteries. Nat. Commun..

[2] Tang S., Guo W., Fu Y. (2021). Advances in
Composite Polymer Electrolytes for Lithium
Batteries and Beyond. Adv. Energy Mater..

[3] Enayati-Gerdroodbar A., Eliseeva S. N., Salami-Kalajahi M. (2023). A Review on the Effect of Nanoparticles/Matrix
Interactions on the Battery Performance of Composite Polymer Electrolytes. J. Energy Storage.

[4] Zhang J., Tian Y., Jia X., Ding S., Wu H. B., Su Y. (2025). Beyond Composition:
Optimizing Ion Transport in Solid-State Composite
Polymer Electrolytes through Pathway Engineering. J. Am. Chem. Soc..

[5] Bocharova V., Chen X. C., Jeong S. P., Zhou Z., Sacci R. L., Keum J. L., Gainaru C., Rahman M. A., Sahori R., Sun X. G., Cao P., Westover A. (2023). Single Ion Conduction
Hairy Nanoparticle Additive to Improve Cycling Stability of Solid
Polymer Electrolytes. ACS Appl. Energy Mater..

[6] Sethi G. K., Jung H. Y., Loo W. S., Sawhney W., Park M. J., Balsara N., Villaluenga I. (2019). Structure
and Thermodynamics of Hybrid
Organic-Inorganic Block Copolymers with Salt. Macromolecules.

[7] Nikolakakou G., Pantazidis C., Sakellariou G., Glynos E. (2026). Thermal Properties
and Ion Redistribution in Nanostructured Single-Ion Polymer Electrolyte
Blends Composed of Polyanionic Miktoarm Stars. ACS Macro Lett..

[8] Ueno K., Hata K., Katakabe T., Kondoh M., Watanabe M. (2008). Nanocomposite
Ion Gels Based on Silica Nanoparticles and an Ionic Liquid: Transport,
Viscoelastic Properties and Microstructure. J. Phys. Chem. B.

[9] Choudhury S., Agrawal A., Kim S. A., Archer L. A. (2015). Self-Suspended Suspensions
of Covalently Grafted Hairy Nanoparticles. Langmuir.

[10] Barbosa J.
C., Gonçalves R., Costa C. M., de Zea Bermudez V., Fidalgo-Marijuan A., Zhang Q., Lanceros-Méndez S. (2021). Metal-Organic
Frameworks and Zeolite Materials as Active Fillers for Lithium-Ion
Battery Solid Polymer Electrolytes. Mater. Adv..

[11] Jamal H., Khan F., Kim J. J., Kim E., Lee S., Kim J. H. (2024). Compact Solid Electrolyte Interface Realization Employing
Surface-Modified Fillers for Long-Lasting, High-Performance All-Solid-State
Li-Metal Batteries. Small.

[12] Kim S., Jamal H., Khan F., Al-Ahmed A., Abdelnaby M. M., Al-Zahrani A., Chun S. E., Kim J. H. (2024). Achieving High Durability
in All-Solid State Lithium Metal Batteries Using Metal-Organic Framework
Solid Polymer Electrolytes. J. Mater. Chem.
A Mater..

[13] Asua J. M. (2004). Emulsion
Polymerization: From Fundamental Mechanism to Process Developments. J. Polym. Sci..

[14] Nunes J., Asua J. M. (2012). Theory-Guided Strategy for Nanolatex Synthesis. Langmuir.

[15] Bilgin S., Tomovska R., Asua J. M. (2017). Effect
of Ionic Monomer Concentration
on Latex and Film Properties for a Surfactant-Free High Solids Content
Polymer Dispersions. Eur. Polym. J..

[16] Porcarelli L., Shaplov A. S., Salsamendi M., Nair J. R., Vygodskii Y. S., Mecerreyes D., Gerbaldi C. (2016). Single-Ion Block Copoly­(Ionic Liquid)­s
as Electrolytes for All-Solid State Lithium Batteries. ACS Appl. Mater. Interfaces.

[17] Porcarelli L., Sutton P., Bocharova V., Aguirresarobe R. H., Zhu H., Goujon N., Leiza J. R., Sokolov A., Forsyth M., Mecerreyes D. (2021). Single-Ion
Conducting Polymer Nanoparticles as Functional
Fillers for Solid Electrolytes in Lithium Metal Batteries. ACS Appl. Mater. Interfaces.

[18] Gallastegui A., Del Olmo R., Criado-Gonzalez M., Leiza J. R., Forsyth M., Mecerreyes D. (2024). Printable
Single-Ion Polymer Nanoparticle Electrolytes
for Lithium Batteries. Small Science.

[19] Stigliano P. L., Gallastegui A., Smith T. H., O’Dell L., Mecerreyes D., Pozo-Gonzalo C., Forsyth M. (2025). Gel Polymer Electrolytes
Based on Sulfonamide Functional Polymer Nanoparticles for Sodium Metal
Batteries. Phys. Chem. Chem. Phys..

[20] Herranz
Berzosa A., Gallastegui A., Lingua G., Mantione D., Villaluenga I., O’Dell L., Forsyth M., Mecerreyes D. (2025). Lithium Single-Ion
Copolymer Nanoparticle Design for Salt-Free Quasi-Solid-State Electrolytes. ACS Appl. Polym. Mater..

[21] Liu X., Mao W., Gong J., Liu H., Shao Y., Sun L., Wang H., Wang C. (2023). Enhanced Electrochemical Performance
of PEO-Based Composite Polymer Electrolyte with Single-Ion Conducting
Polymer Grafted SiO2 Nanoparticles. Polymers
(Basel).

[22] Leslie F. J., Stakem K. G., Gregory G. L. (2025). The Sustainable
Potential of Single-Ion
Conducting Polymers. ChemSusChem.

[23] Olmedo-Martinez J. L., Del Olmo R., Gallastegui A., Villaluenga I., Forsyth M., Müller A. J., Mecerreyes D. (2024). All-Polymer
Nanocomposite as Salt-Free Solid Electrolyte for Lithium Metal Batteries. ACS Polymers Au.

[24] Olmedo-Martinez J. L., Lingua G., Unanue L., Krol M., Ruokolainen J., Müller A. J., Mecerreyes D. (2025). Boosting Ionic
Conductivity by Ordering
Nanoparticles within All-Polymer Poly­(Ethylene Oxide) (PEO) Nanocomposites. ACS Polymers Au.

[25] Makhlooghiazad F., O’Dell L. A., Porcarelli L., Forsyth C., Quazi N., Asadi M., Hutt O., Mecerreyes D., Forsyth M., Pringle J. M. (2022). Zwitterionic Materials
with Disorder
and Plasticity and Their Applications as Non-Volatile Solid or Liquid
Electrolytes. Nat. Mater..

[26] Garcia Y., Porcarelli L., Zhu H., Forsyth M., Mecerreyes D., O’Dell L. A. (2023). Proving Disorder and Dynamics in
Composite Electrolytes
of an Organic Ionic Plastic Crystal and Lithium Functionalized Acrylic
Polymer Nanoparticles. J. Magn. Reson. Open.

[27] Makhlooghiazad F., Porcarelli L., Mecerreyes D., Forsyth M., O’Dell L. A., Pringle J. M. (2024). Composite Lithium Conducting Solid Electrolytes Based
on Zwitterionic Plastic Crystals and Polymer Nanoparticles. Mater. Adv..

